# Heavy Metal-Induced Genotoxic Damage in *Chelon auratus*: Evidence from a Coastal Gulf Ecosystem

**DOI:** 10.3390/toxics14080732

**Published:** 2026-08-19

**Authors:** Cemal Turan, Aysegul Ergenler, Zeynep Ayad Koç, Funda Turan

**Affiliations:** 1 Molecular Ecology, Genomics and Computational Biology Laboratory, Department of Molecular Biology and Genetic, Faculty of Engineering and Natural Sciences, Istanbul Atlas University, 34403 İstanbul, Türkiye; 2 Department of Basic Sciences, Istanbul Nişantaşı University, 34398 Istanbul, Türkiye; 3 Faculty of Marine Science and Technology, Iskenderun Technical University, 31200 Hatay, Türkiye

**Keywords:** DNA damage, metals, comet assay, biomonitoring

## Abstract

Estuarine and river-influenced coastal ecosystems are recognized as important sinks and channels for transfer of heavy metals into the marine environment. Continuous intake of metal pollutants could create chronic exposure situations, perhaps leading to molecular and cellular damage to resident biota, even if environmental concentrations are within regulatory limits. Thus, the incorporation of molecular and genotoxicity biomarkers into environmental monitoring programs has received growing interest, as changes in gene expression are among the earliest detectable responses to pollutant stress and may precede genotoxic effects, including DNA damage, at higher or prolonged levels of contaminant exposure. The present study aimed to determine the levels of heavy metals in the coastal zone where the Deliçay River flows into the Gulf of Iskenderun in the extreme northeastern Mediterranean Sea, Türkiye, and to investigate the genotoxic effects in the euryhaline ray-finned fish golden grey mullet (*Chelon auratus*). In this study, seasonal water samples (*n* = 3 per site per season) and *C. auratus* specimens (*n* = 10 per site per season; total *n* = 80) were collected from a reference site and the Deliçay estuary. Water samples were analyzed for metals (cadmium (Cd), chromium (Cr), iron (Fe), lead (Pb), and zinc (Zn)) and fish samples were analyzed with the micronucleus (MN) test for determining nuclear abnormalities and the comet test for DNA damage levels. The concentrations of Fe, Zn, and Pb in seawater exceeded the Criterion Continuous Concentration (CCC) thresholds during the summer and autumn seasons, as well as in terms of annual mean values, indicating a potential chronic ecological risk to marine organisms. From the results of the micronucleus test performed in the present study, the highest MN frequencies (10.16 ± 0.15%) and other erythrocytic nuclear anomalies [kidney-shaped (10.36 ± 0.32%), binucleated (14.20 ± 0.10%), notched (14.63 ± 0.20%), lobed (15.43 ± 0.11%), and budded (15.33 ± 0.15%)] were found along the studied coastal zone in summer season. Results of the comet test, supporting the micronucleus test results, showed the highest percentages of DNA damage determined in all seasons in the gill and liver tissues of fish sampled in the studied coastal zone. This study is the first to evaluate the effects of heavy metal-induced genotoxic stress on ecological integrity in this coastal zone using a biomarker-based approach, and the results underscore the need for comprehensive environmental monitoring and pollution reduction strategies to protect ecosystem health.

## 1. Introduction

One of the most serious environmental problems is anthropogenic sources of heavy metals in aquatic ecosystems, which arise from industrial activities, mining, energy production, port activities, agricultural drainage, domestic waste discharges and heavy ship traffic. Heavy metals are non-biodegradable, persist in the environment for long periods, and tend to bioaccumulate and be biomagnified through trophic levels, leading to severe toxic effects on aquatic organisms. Specifically, elevated environmental levels of metals such as cadmium (Cd), lead (Pb), mercury (Hg), chromium (Cr), and nickel (Ni) interfere with cellular metabolism, enhance the production of reactive oxygen species, and trigger oxidative stress mechanisms, thus resulting in genotoxic effects such as DNA strand breaks, chromosomal abnormalities, and mutations [1,2,3]. Thus, genotoxicity induced by heavy metals is a major biological risk factor threatening the physiological integrity of aquatic organisms. The Gulf of Iskenderun is one of the coastal areas of the Eastern Mediterranean in which the most intensive industrial activities are performed. This ecosystem is particularly sensitive to anthropogenic disturbances and has been under environmental pressure for many years from iron and steel factories, thermal power plants, fertilizer and petrochemical facilities, port operations and intensive maritime transport. Anthropogenic activities, especially along the Payas, Dörtyol and Iskenderun coasts of the gulf, have resulted in increased heavy metal accumulation in the water and sediment environment. The studies carried out in the last decade showed that metals such as cadmium (Cd), lead (Pb), chromium (Cr), copper (Cu), iron (Fe), zinc (Zn), and nickel (Ni) have reached high concentrations in the environmental matrices of the gulf [2,4,5]. Turan et al. [2] reported high DNA damage frequency (94.00 ± 4.35%) of *Chelon auratus* in the Payas coast of Iskenderun bay compared to the reference site because of pollutants, namely high levels of Cd (1.61 ± 0.11 μg g^−1^), Pb (5.06 ± 0.43 μg g^−1^), Fe (249.49 ± 74.83 μg g^−1^) and Cu (57.62 ± 3.61 μg g^−1^) accumulation detected in *C. auratus* that exceeded the maximum levels allowed by national and international safety limits. A significant positive correlation between Cd, Pb, Hg, Cr, Fe, Zn and Cu accumulations and DNA damage parameters was reported in their study. In addition, the sediment structure of the region has the potential to retain pollutants for a long period and the semi-enclosed hydrodynamic nature of the gulf favors the accumulation of pollutant load in the system [6]. Due to the intensity of industrial activities, the Dörtyol district and its vicinity on the eastern coast of the Gulf are among the places where environmental pollution is most clearly observed. One of the important surface water resources carrying pollutants from industrial areas, residential centers and agricultural lands to the Gulf of Iskenderun is the Deliçay River in this region. This river increases the transport of particulate matter containing heavy metals and dissolved pollutants, especially during rainy periods, and increases the exposure of aquatic organisms in the coastal ecosystem to these pollutants. It has been reported that heavy metals inhibit enzymatic systems in aquatic organisms, disrupt the integrity of cellular membranes and cause irreversible damage to genetic material [1,7]. In recent years, there has been wide use of genotoxicity biomarkers to evaluate the biological effects of environmental pollutants. The comet assay is considered one of the most sensitive methods for the detection of DNA strand breaks and genetic damage at the cellular level. Heavy metal exposure induces oxidative stress, which is known to cause DNA damage and leads to irreversible loss of cellular function. Thus, genotoxicity analysis is one of the important biological monitoring methods in environmental risk assessment studies in aquatic environments [2,8]. Fish species are often used as biomonitoring organisms because they can reflect the chemical characteristics of their environment and bioaccumulate pollutants in their tissues. In particular, the euryhaline ray-finned fish golden grey mullet (*C. auratus*) is considered an essential indicator species for the recognition of biological effects of heavy metal pollution, due to its bentopelagic feeding features and its direct contact with pollutants in the sediment and the water column [2].

To date, the only genotoxicological investigations addressing metal pollution in the Iskenderun Gulf, northeastern Mediterranean, have been conducted by our research team [2,9,10,11]. However, comprehensive literature surveys have revealed that no previous study has investigated the ecotoxicological status of the coastal zone as a result of the Deliçay River’s discharge into the Iskenderun Gulf. Previous environmental studies conducted in the region have consistently identified Cd, Cr, Fe, Pb, and Zn as the predominant heavy metals associated with anthropogenic activities [2,4,5]. Therefore, these metals were selected because they represent the major contaminants reported in the study area and are known to pose potential ecological risks to aquatic organisms. We hypothesized that *C. auratus* inhabiting the Deliçay coastal zone would exhibit increased genotoxic responses associated with chronic exposure to these metals. Accordingly, the objectives of this study were to determine the concentrations of Cd, Cr, Fe, Pb, and Zn in coastal waters and to evaluate genotoxic responses in *C. auratus* using the micronucleus and comet assays. The findings are expected to contribute to environmental risk assessment and biomonitoring strategies for the Iskenderun Gulf coastal ecosystem.

## 2. Materials and Methods

### 2.1. Study Area

#### 2.1.1. Overview of Study Area

**Figure 1:**
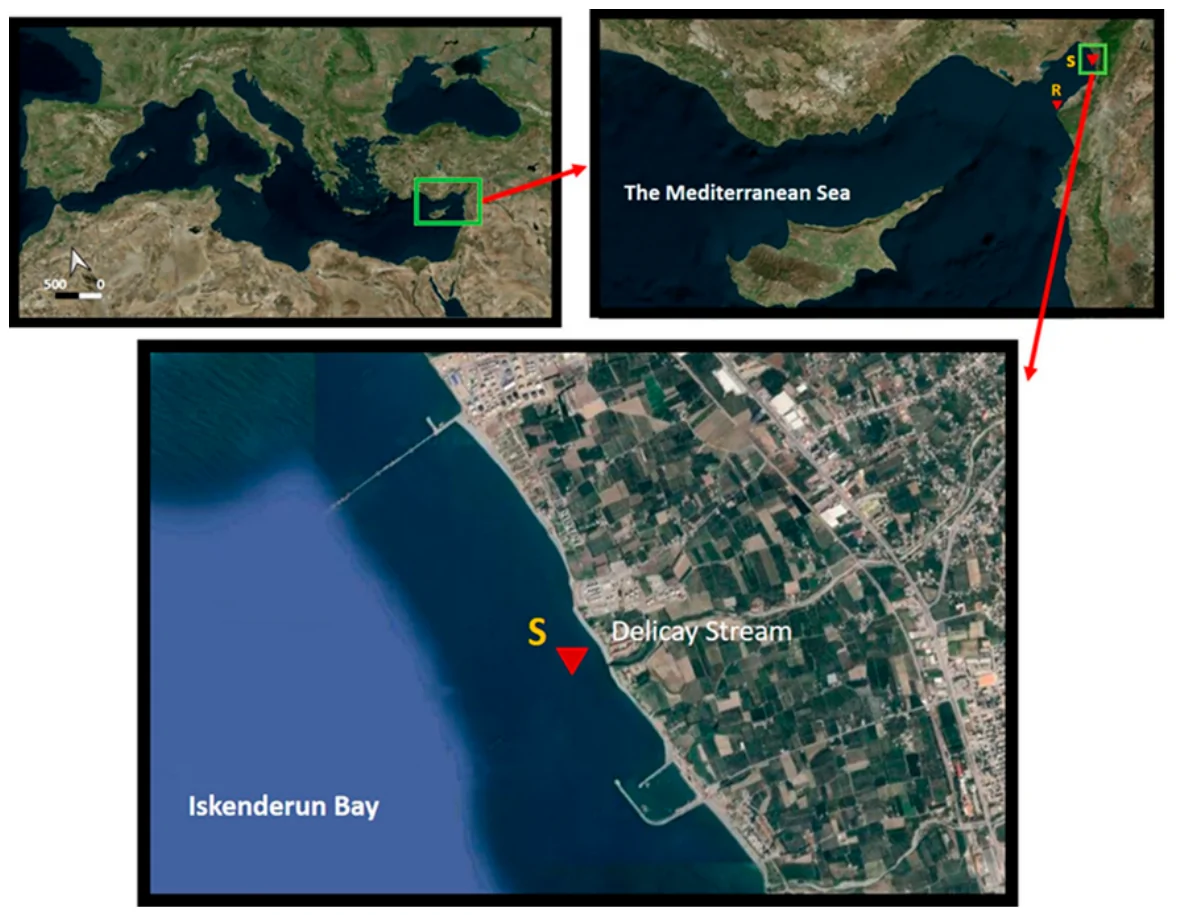
Sampling map of the study area. The coastal area where the Deliçay Stream flows into the Iskenderun Gulf. The sampling (S) and reference (R) sites from Konacık located at the Gulf of Iskenderun in the Northeastern Mediterranean.

This study was conducted in the coastal area where the Deliçay Stream, one of the important freshwater sources flowing into the Gulf of Iskenderun, flows into the gulf (36°50′25″ N, 36°12′04″ E) (Figure 1).

Located on the northeastern Mediterranean coast of Turkey, the Gulf of Iskenderun is one of the important coastal ecosystems under high anthropogenic pressure due to intensive fishing activities, as well as port operations, industrial complexes, oil loading facilities, and increasing maritime traffic [2]. Rapid industrialization and population growth around the gulf increase the risk of pollution to aquatic environments in the region. Furthermore, recent studies have revealed that floating marine debris and various pollutant inputs in the Gulf of Iskenderun exert pressure on the ecosystem. The Deliçay Stream, chosen as the study area, and the coastal region where the stream flows into the gulf, contain intensive agricultural activities and industrial waste inputs. Citrus processing plants, operating in conjunction with the widespread citrus production and trade in the region, use large amounts of water, and the resulting wastewater is discharged into the stream system through various routes. Recent studies conducted on the streams flowing into the Gulf of Iskenderun show that the Deliçay Stream is under pollution pressure in terms of physicochemical parameters and carries a risk of microbiological contamination [12]. 

The Konacık region was selected as the reference station for this study, and water and fish samples will be collected from both sampling sites. Due to the absence of industrial facilities in this region, it is free from industrial pollution. Additionally, because it is a mountainous area with low population density and the gulf is an open area, it has been evaluated as a reference station. 

#### 2.1.2. Collecting Samples and Procedure

Sampling was conducted seasonally in March (spring), June (summer), September (autumn), and December (winter) 2024 at two coastal locations, namely a reference site (R) and a sampling site (S) influenced by the Deliçay River discharge. During each sampling campaign, three independent water samples (*n* = 3) were collected from each site for physicochemical and heavy metal analyses. In addition, ten *C. auratus* individuals (*n* = 10) were collected from each site during each season for biomarker analyses. In total, 24 water samples (12 per site) and 80 fish specimens (40 per site) were included in the study.

Dark-colored polyethylene (PE) bottles with a volume of 1000 mL, previously cleaned with a 10% HNO_3_ solution and deionized water, were used for sampling. Before sampling, the bottles were rinsed with a small amount of the sampled water and then completely filled with running water. The collected water samples were transported to the laboratory under cold chain conditions at +4 °C until analysis. Some basic physicochemical parameters of water quality were measured in situ during sampling. Water temperature (°C), pH, and dissolved oxygen (mg/L) values were determined using portable YSI brand oxygen meters and pH meters. Salinity, electrical conductivity, and total dissolved solids (TDS) measurements were performed using a portable YSI type salinity/conductivity meter.

*C. auratus* was used as the biological material in the study. This species is widely used as a bioindicator organism in studies of heavy metal accumulation and environmental stress [2,9]. Fish samples were obtained using the net casting method in cooperation with local fishermen. Sampling was conducted seasonally over a one-year period, and a total of 80 fish specimens were examined as a result of field studies carried out in four different periods. Care was taken to ensure that the sampled individuals represented different gender and size groups. The biometric characteristics of the *C. auratus* specimens used in the present study are summarized in Table 1. Water samples were collected from the same coastal locations where *C. auratus* specimens were captured. Since *C. auratus* is a resident coastal species inhabiting shallow nearshore waters, the analyzed water samples were considered representative of the environmental conditions to which the fish were chronically exposed. This sampling strategy enabled the relationship between heavy metal concentrations in seawater and the observed genotoxic responses in fish to be evaluated.

### 2.2. Metal Analysis

#### 2.2.1. Preparation of Water Samples

Water samples brought to the laboratory were filtered with blue tape filter paper and pre-processed within approximately 72 h, or if analysis was not to be started, the pH value was adjusted to 2–3 with a 1 + 1 HNO_3_ solution and the samples stored for a longer period. For heavy metal analysis, 100 mL of each water sample was transferred into a 250 mL digestion vessel and acidified by adding 5 mL of 55% HNO_3_, corresponding to an approximate final concentration of 5% (*v*/*v*) HNO_3_. The acidified samples were heated on a hot plate and concentrated to approximately 20 mL. Subsequently, 5 mL of 55% HNO_3_ and 10 mL of 70% HClO_4_ were added, and digestion was continued until the evolution of brown fumes ceased and white fumes appeared. After cooling to room temperature, the digested samples were diluted to 100 mL with distilled water prior to elemental analysis [13]. Water sample preparation prior to ICP-MS analysis was performed according to the acid digestion procedure described in APHA Standard Methods (Method 3030E) prior to ICP-MS analysis [14]. Water samples were collected seasonally from both the reference (R) and sampling (S) sites in March, June, September, and December 2024. During each sampling campaign, three replicate water samples (*n* = 3) were collected from each site, resulting in a total of 12 water samples from each site (24 samples overall). 

#### 2.2.2. Measurements of Metals

Metal measurements in water were performed at the Iskenderun Technical University Science and Technology Research and Application Center (İSTE-BTM) using Analytik Jena/Plasmaaquant ICP-MS Elite (Jena, Germany). As a result of the measurements, the values of Cd, Cr, Fe, Pb, and Zn in the samples were calculated in µg L^−1^ using mathematical methods. Standards were prepared daily from the metal-free Merck brand ICP Standard (Multielement-IV), and the analyses were performed in parallel. Detection limits according to ICP-MS Elite for Water Samples: cadmium: 0.0008 ppb; lead: 0.0016 ppb; chromium: 0.0097 ppb; Fe: 1.4756 ppb; Zn: 0.1331 ppb.

Quality assurance/quality control (QA/QC) procedures were implemented throughout the ICP-MS analysis. Instrument calibration was performed using multi-element standard solutions prior to sample analysis, and analytical performance was verified using a 10 µg/L quality control (QC) standard. The measured QC concentrations were in close agreement with the nominal values. The relative standard deviation (RSD) values for the QC standard were 0.6% (Cd), 0.5% (Cr), 4.3% (Fe), 1.3% (Pb), and 1.3% (Zn), indicating satisfactory analytical precision. Method blanks were also analyzed during the analytical sequence to monitor potential contamination and background signals.

### 2.3. Genotoxicity Tests

#### 2.3.1. Micronucleus (MN) Test

Analyses aimed at determining nuclear abnormalities occurring in *C. auratus* erythrocytes were conducted at the Genotoxicology Laboratory of the Faculty of Marine Sciences and Technology, Iskenderun Technical University. Fish samples, collected live from local fishermen during field studies, were transported to the laboratory the same day in air-pressurized containers under appropriate conditions. Sedation was administered to the fish to minimize stress during transport with clove oil (5 mg L^−1^) [15]. For micronucleus (MN) analysis, blood samples were taken from the heart region of live fish using a heparinized syringe. Following blood collection, the fish were humanely euthanized with an overdose of quinaldine sulphate (5 mg L^−1^; Sigma Chemical Company, Steinheim, Germany). Peripheral smear preparations were made from the blood samples, and after drying at room temperature, the preparations were fixed and subjected to Giemsa staining. After staining, the preparations were dried again, and all preparations, including positive and negative controls, were coded before microscopic examination. The prepared slides were examined using a light microscope (Olympus BX53, Olympus Corporation, Tokyo, Japan) at 1000× magnification under oil immersion. In microscopic evaluations, at least 1000 erythrocyte cells were counted for each individual, and the obtained data were analyzed according to the criteria in [16]. Micronuclei were identified according to established morphological criteria. Briefly, micronuclei were round or oval nuclear bodies with a diameter of less than one-third (<33%) of the main nucleus, exhibiting staining intensity and texture similar to those of the main nucleus, and clearly separated from it. Only well-preserved, intact, non-overlapping erythrocytes were evaluated for micronucleus and other nuclear anomalies. Morphological nuclear anomalies were examined on erythrocyte cells in peripheral blood smears. Evaluations were performed under five main groups according to the classification reported by [16]: kidney nucleus, notched nucleus, budding nucleus, lobbed nucleus, and binucleus. A total of 1000 cells were counted in each preparation, and morphological nuclear anomalies were recorded. To minimize observer bias, all microscope slides were identified only by numerical codes during microscopic examination. Micronucleus and nuclear abnormality scoring was performed by a single experienced observer using identical scoring criteria for all slides.

#### 2.3.2. Comet Assay

Single cell gel electrophoresis was performed according to the method reported by Singh et al. [8]), with minor modifications. Briefly, gill and liver tissues were washed twice with ice-cold phosphate-buffered saline (PBS; Ca^2+^- and Mg^2+^-free), followed by the addition of cold homogenization buffer to obtain a single-cell suspension. The cell suspension was centrifuged at 3000 rpm for 5 min at 4 °C, and 10 μL of the resulting cell suspension was mixed with 100 μL of 0.7% low-melting-point agarose (LMPA). Subsequently, 100 μL of the mixture was spread onto normal-melting-point agarose (NMPA)-coated slides and allowed to solidify. The slides were immersed in lysis solution overnight at 4 °C, followed by incubation in alkaline electrophoresis buffer for 20 min at 4 °C. Electrophoresis was then performed under alkaline conditions at 24 V and 300 mA for 40 min at 4 °C. Finally, the slides were stained with 100 μL ethidium bromide (10 μg mL^−1^) prior to microscopic evaluation, and the preparations were examined under a fluorescence microscope (Carl Zeiss Axiostar Plus, AzoMaterials, Jena, Germany) at ×400 magnification. The criteria defined by [17]) were used to evaluate DNA damage. A total of 100 cells were analyzed from each sample (gill and liver tissue), and nucleoids were graded from 0 to 4 according to tail density. In this classification, “0” indicates the absence of any tail formation, while “4” indicates that the majority of DNA is located in the tail region. Based on the tail test results, DNA damage percentage (%DF), arbitrary units (AUs), and genetic damage index (GDI) were calculated according to the methods reported by [17,18].

### 2.4. Statistical Analysis

Before statistical evaluation of the obtained data, normality distribution was determined using the Shapiro–Wilk test, and homogeneity of variance was determined using the Levene test. One-way analysis of variance (ANOVA) was applied to evaluate differences between groups, and the statistical significance level was accepted as *p* < 0.05 [19]. All statistical analyses were performed using IBM SPSS Statistics v21 and R-Studio software (Version 4.5.3).

## 3. Results

### 3.1. Physico-Chemical Parameters

Table 2 shows the seasonal physicochemical parameters in the sampling and reference sites over a year.

### 3.2. Trace Metals

Trace concentrations of metals such as Cd, Cr, Fe, Pb, and Zn in water samples taken from sampling and reference points are presented in Table 3. 

The highest metal concentrations were generally recorded at the sampling site, particularly during summer and autumn. Among the analyzed metals, Fe, Zn, and Pb exhibited the greatest spatial differences between the sampling and reference sites. These differences were also found to be statistically significant (*p* < 0.001). However, this pattern was not consistent across all seasons. During winter, Cd, Cr, Fe, and Pb concentrations were significantly higher at the reference site than at the sampling site, whereas Zn remained higher at the sampling site. While most measured concentrations complied with the TEG and USEPA water quality criteria, Pb exceeded the TEG Continuous Concentration Criterion (CCC) during the summer, autumn, and annual assessment, whereas Fe and Zn exceeded the TEG CCC values during specific sampling periods. These findings indicate that the coastal area influenced by the Deliçay River receives a continuous metal input despite the generally acceptable physicochemical water quality. The highest metal concentration in the water samples from the sampling site was iron. The highest iron concentration was found in the autumn months (60.66 ± 7.41 µg L^−1^), and the lowest in the winter months (25.27 ± 4.49 µg L^−1^). Seasonal and annual assessments revealed that metal concentrations in the water, from highest to lowest, were as follows: Fe > Zn > Pb > Cd > Cr (Table 3).

### 3.3. Micronucleus Test

Within the scope of the research, the results of micronucleus and chromosomal abnormalities in the peripheral blood cells of *C. auratus*, sampled seasonally over the course of one year, are presented in Table 4.

Throughout all seasons, markedly elevated micronucleus (MN) frequency values were detected in the erythrocytes of *C. auratus* obtained from the sampling region (the Dörtyo–Deliçay region of the Iskenderun Gulf) in comparison to those taken from the reference site (*p* < 0.01; Table 4). Statistically significant differences (*p* < 0.05) were observed between stations for both seasonal and annual variations.

**Figure 2:**
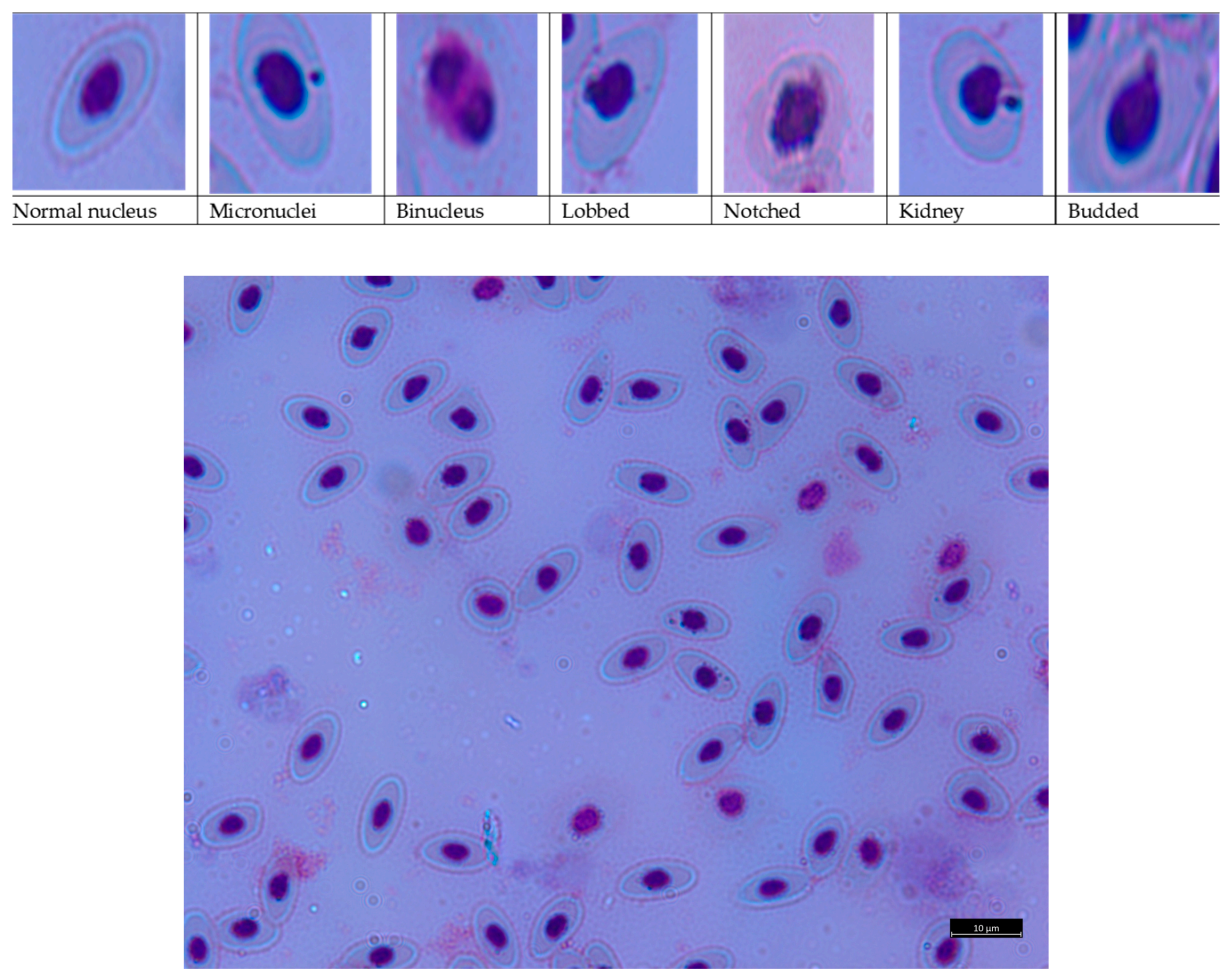
Photomicrographs showing cells with micronuclei and other nuclear anomalies in peripheral blood erythrocytes of *C. auratus*. Scale bars represent 10 μm in all panels.

The highest MN frequencies and other erythrocytic nuclear anomalies (kidney-shaped, binucleated, cotillated, lobed, and budded) were detected at the Dörtyol–Deliçay region of the Iskenderun Gulf in all seasons. As expected, the lowest MN frequencies were detected in the erythrocytes of fish from the reference site. MN frequencies peaked in the summer season (10.16 ± 0.15%), while they declined to minimum values in the autumn season (3.40 ± 0.26%) (Table 4). Representative photomicrographs of micronuclei (MN) observed in *C. auratus* are presented in Figure 2.

### 3.4. Comet Assay

**Figure 3:**
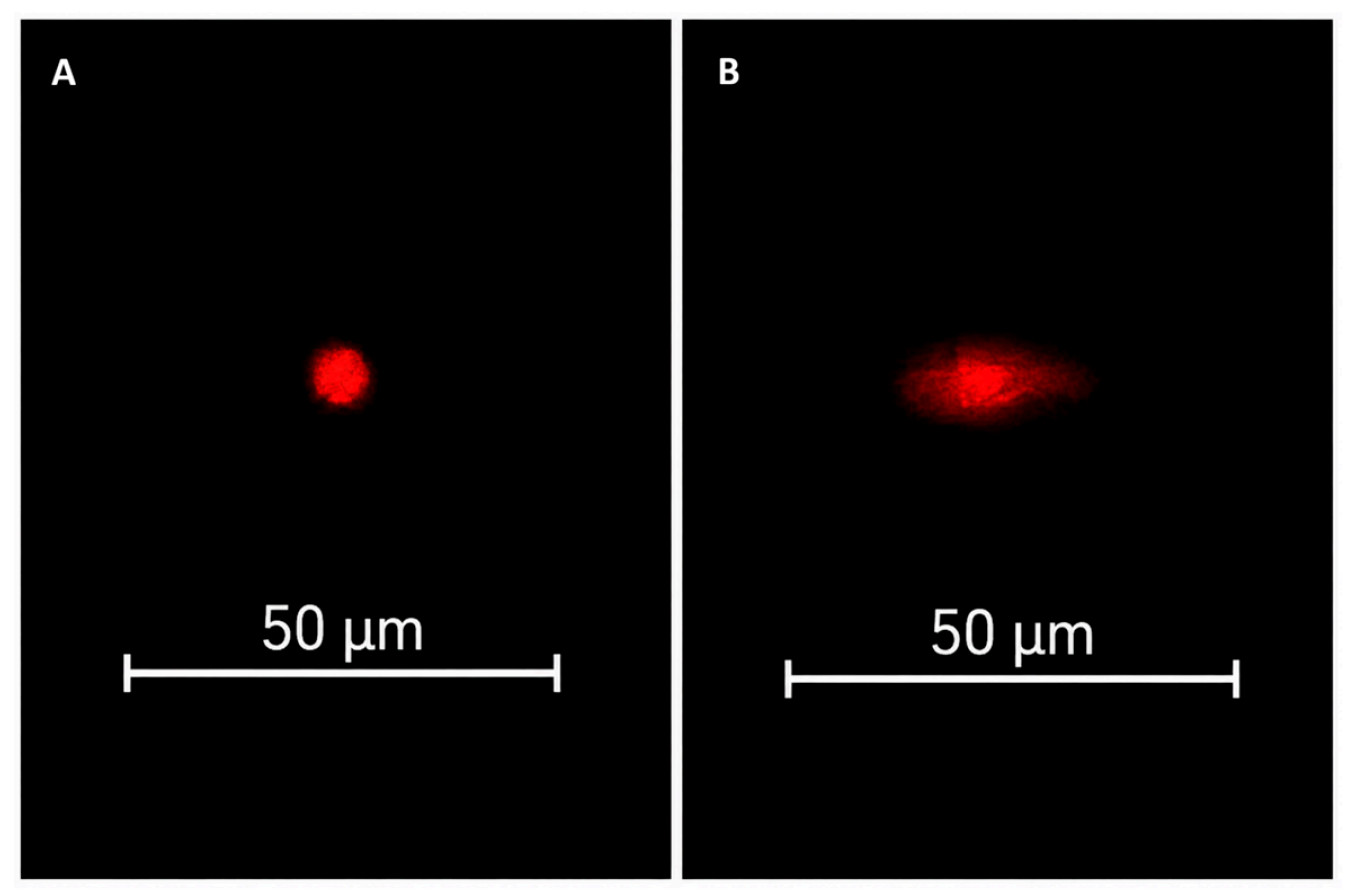
Comet assay showing DNA damage (no damage (**A**) and damaged cells (**B**)) in liver cells of *C. auratus*.

Within the scope of the research, damage frequency (DF %), arbitrary units (AUs) and damage index (DI %) of DNA damage in *C. auratus* sampled from sampling and reference sites were evaluated through the comet assay, and the results are given in Table 5. Representative comet assay images corresponding to different cells with DNA damage are provided in Figure 3.

Throughout all seasons, the highest DNA damage percentages were seen in the Dörtyol–Deliçay region of the Iskenderun Gulf (Table 5). As expected, the lowest levels of DNA damage were seen in the gill and liver tissues of fish from the reference site. Both seasonal and annual inter-station differences were found to be statistically significant (*p* < 0.05). 

The highest percentage of DNA damage in gill tissues was recorded in the autumn season (50.00 ± 3.61%), whereas the minimum values were observed in the winter season (32.00 ± 2.65%) (Table 4). Similarly, peak damage in liver tissues occurred in the autumn season (45.67 ± 7.23%), while minimum levels were observed in the winter season (28.00 ± 1.01%). In all seasons and stations, relatively higher levels of DNA damage were detected in the gill tissue compared to the liver, and this difference is statistically significant (*p* < 0.05). In the annual analyses of DNA damage percentage (DF %), AU, and GDI (%) parameters in *C. auratus* samples obtained from sampling and reference sites, statistically highly significant differences were observed (*p* < 0.001) (Table 5).

## 4. Discussion

This study is the first to demonstrate significant spatial differences in both heavy metal concentrations and genotoxic biomarkers between the Deliçay coastal region and a reference region in the Northeast Mediterranean. Specifically, Fe, Zn, and Pb showed the most pronounced increases in the sampling region, while fish collected from this region had significantly higher frequencies of micronucleus, erythrocyte nuclear abnormalities, and DNA damage compared to those collected from the reference region. These findings suggest that the coastal region affected by the Deliçay River is continuously exposed to anthropogenic metal inputs, and that biomarker-based approaches provide valuable information for assessing the biological consequences of environmental pollution. The following discussion interprets these findings by relating them to regional pollution sources, environmental quality criteria, and potential underlying mechanisms of metal-induced genotoxicity.

The coastal seawater quality metrics identified in this study region were consistent with the temperature, pH, and salinity thresholds established by [23] the 2015 Turkish Environmental Guiding Principles Coastal Water Quality Criteria. Consequently, while the assessed physicochemical parameters conform to quality standards, it is essential to recognize that estuarine and river-influenced coastal ecosystems are sensitive environments that require thorough evaluation regarding the chronic effects of genotoxic pollutants.

In each of the environmental samples examined in this study, the analyzed metals (Cd, Cr, and Pb) are among the top-ranked on the International Agency for Cancer Research’s (IARC) list of priority carcinogenic metals [24]. The other two metals (Fe and Zn) are consistently present in high concentrations in the aquatic ecosystem of the Iskenderun Gulf due to the intensive iron and steel industry [2]. When seasonal and annual values were examined, the concentrations of Cd, Cr, Fe, Pb, and Zn in the water at the sampling point were higher than those at the reference point, except for the autumn Cd concentration, and the toxic metal values of the reference region were found to be statistically significant. Overall, the concentrations of Fe, Zn, and Pb in seawater exceeded the criterion continuous concentration (CCC) thresholds during the summer and autumn seasons, as well as in terms of annual mean values, indicating a potential chronic ecological risk to marine organisms. In contrast, metal concentrations measured at the reference site remained below all guideline values throughout the study period. These findings suggest that the impacted area is subjected to sustained metal inputs, whereas the reference site maintains relatively unpolluted conditions and can therefore be considered representative of the regional background levels.

Iron and steel industry activities in the interior of the gulf, particularly in the area close to the study site—process water, coke production, sintering, blast furnace and steel casting—are the main cause of the increase in Fe concentrations in the seawater. High levels of Fe^2+^ and Fe^3+^ ions, cyanide complexes, and metal-containing wastes are released into coastal ecosystems due to acidic drainage and wash waters from cooling towers and slag storage areas, or carried into coastal zones by surface runoff [25]. Under neutral pH and high oxygen conditions, Fe entering seawater rapidly transforms into Fe(III) oxyhydroxide flocs, precipitating and accumulating in benthic habitats, suffocating primary producers and aquatic invertebrates and bioaccumulating through trophic transfer [26]. The Gulf of Iskenderun is one of the significant industrial regions of Turkey, affected by iron and steel mills, metal processing industries, port activities, energy producing facilities and dense urban settlements. Such high anthropogenic activities lead to the transport and buildup of harmful heavy metals including Pb to the coastal marine environment. The heavy metal load in the gulf was determined to be mostly from industrial discharges, ship traffic, and land-based inputs [9,27,28,29]. The examinations of seawater show that lead concentrations increase particularly during summer and autumn seasons. This is linked to limited rainfall, increased evaporation, metal concentration in the water column and the continuance of industrial activity. Lead concentrations pose the risk of chronic toxicity to marine organisms both in summer and autumn periods when yearly average values are above the criterion continuous concentration (CCC) values determined by the US Environmental Protection Agency (USEPA) for the protection of marine life. The elevated Pb concentrations detected in the present study are consistent with previous investigations conducted in the Iskenderun Gulf, indicating that Pb contamination represents a persistent environmental concern in this coastal ecosystem. Lead is recognized as a genotoxic metal capable of inducing oxidative stress and DNA damage in aquatic organisms. However, although Pb concentrations exceeded the Turkish Environmental Guidelines (TEG) criterion continuous concentration (CCC) during some sampling periods, they remained within the U.S. EPA guideline values. Therefore, the genotoxic responses observed in the present study should not be attributed solely to Pb exposure. Under natural environmental conditions, fish are chronically exposed to complex mixtures of contaminants, and the combined effects of multiple heavy metals and other pollutants are more likely to contribute to the observed increases in micronucleus frequency and DNA damage than exposure to a single metal alone. Furthermore, these findings were also in agreement with the investigations of [2,11,30,31], who showed that mullets can collect hazardous metals in their body tissues due to their feeding habits and lifestyles. Thus, mullets can be employed as reliable biological monitoring tools in coastal environments.

From the results of the micronucleus test performed in our study, the highest MN frequencies and other erythrocytic nuclear anomalies [kidney-shaped, binucleated, cotillated, lobed and budded] were found along the coastal zone where the Dörtyol–Deliçay stream meets the Iskenderun Gulf in all seasons. Published baseline micronucleus frequencies for healthy *C. auratus* are limited. However, studies conducted on other teleost species generally report low spontaneous micronucleus frequencies under uncontaminated environmental conditions, whereas significantly elevated frequencies are associated with exposure to metal-contaminated habitats. This study also looked into metal-induced DNA strand breaks in various tissues as a biomarker of genotoxicity using the alkaline comet assay. The comet test is widely used for the assessment of genotoxicity due to its high sensitivity [9,32,33], and it has been adopted for the assessment of pollutant-induced DNA damage in many fish species, including mullets [33,34]. The results of the comet test support the micronucleus test results, showing the highest percentages of DNA damage determined in all seasons in the gill and liver tissues of fish sampled in the coastal zone where the Dörtyol–Deliçay stream drains into the Iskenderun Gulf. In particular, the DNA damage levels determined in this study increased during periods of high heavy metal concentrations. Similarly, numerous studies on teleost fish have reported significantly increased DNA damage following exposure to metal pollution, as determined by the comet assay and micronucleus test [2,34,35]. The micronucleus assay has been widely recognized as a reliable biomarker for in situ monitoring of genotoxic pollution in marine teleosts [36]. Likewise, the comet assay is considered one of the most sensitive biomarkers for detecting environmentally induced DNA damage in aquatic organisms, and its combined use with the micronucleus test provides a comprehensive assessment of genotoxic effects [37]. More recently, Kontaş [38] demonstrated that exposure to selected metal(loid)s significantly increased DNA damage and micronucleus frequency in *Cyprinus carpio*, further supporting the applicability of these biomarkers for evaluating metal-induced genotoxicity in teleost fish.

The comet assay was performed using liver and gill tissues because these organs are among the primary target tissues for waterborne contaminants in fish. The gills are continuously exposed to the aquatic environment and constitute the main route of contaminant uptake, whereas the liver plays a central role in the metabolism and detoxification of xenobiotics. Consequently, both tissues are considered highly sensitive indicators of DNA damage induced by environmental pollutants. In contrast, the micronucleus assay was performed on peripheral erythrocytes, as nucleated fish erythrocytes are widely recognized as suitable cells for evaluating chromosomal damage [36,37].

Genotoxicity studies have shown that living in the affected area has non-lethal but physiologically significant genotoxic effects on *C. auratus*, even when trace metal levels are within national and international safety limits [20,21,22]. The comet assay findings clearly showed a statistically significant increase (*p* < 0.001) in DNA strand breakage in gill cells of fish in the sampled site in all seasons. The findings are consistent with recent studies showing seasonal fluctuations in metal bioavailability and oxidative stress potential, especially during the warmer months, when metabolic activity and pollutant uptake in aquatic organisms are intensified [9,36].

The increased frequencies of micronuclei and erythrocytic nuclear abnormalities, together with the elevated DNA damage detected by the comet assay, are likely associated with the combined effects of chronic exposure to multiple heavy metals rather than a single contaminant. Heavy metals are known to induce excessive production of reactive oxygen species (ROS), leading to oxidative stress, DNA strand breaks, oxidative base modifications, and lipid peroxidation. In addition, several metals may interfere with DNA repair mechanisms and disrupt normal chromosome segregation during mitosis, thereby promoting the formation of micronuclei and other nuclear abnormalities. Although the present study was not designed to establish causal relationships between individual metals and genotoxic endpoints, the concurrent occurrence of elevated metal concentrations and increased genotoxic biomarkers at the sampling site is consistent with the mechanisms of metal-induced genotoxicity reported in previous studies. These findings suggest that chronic exposure to metal mixtures may compromise genomic stability and represents a potential ecological risk for coastal fish populations.

When evaluated seasonally, it is seen that changes in DNA damage are not only caused by heavy metal levels, but also that environmental conditions play a significant role. Furthermore, low water levels and increased evaporation due to seasonal variations can lead to higher concentrations of pollutants. This increases the bioavailability of heavy metals, making genotoxic effects more pronounced [35,39]. Studies demonstrating the effect of seasonal differences on genotoxicity report that DNA damage is generally higher during the summer months. For example, a study conducted in the Melet Basin indicated that DNA damage measured in fish erythrocytes reached its highest level during the summer season, and this was associated with increased heavy metal concentrations [40]. This study is consistent with other studies, and our research shows an increase in DNA damage and micronucleus anomalies during the summer season. In addition, seasonal changes in precipitation patterns and stream flow also affect the distribution of heavy metal pollution. Particularly during spring and autumn, the amount of metals transported into the aquatic environment can increase due to agricultural runoff, industrial discharges, and sediment resuspension. The re-transfer of metals deposited in sediments back into the water column increases the exposure levels of fish. Thus, a strong correlation emerges between heavy metal concentrations and DNA damage during certain periods [41]. Therefore, monitoring heavy metal analyses together with genotoxicity biomarkers is of great importance in determining environmental risks in the evaluation of aquatic ecosystems [34,41]. Although seasonal increases in heavy metal concentrations generally coincided with elevated genotoxic responses, the present study was not designed to establish statistical correlations between these variables. Future studies integrating correlation and multivariate analyses would provide a more comprehensive understanding of the relationships between metal exposure and genotoxic biomarkers.

The increased frequencies of micronuclei and DNA damage observed in *C. auratus* should be regarded as early-warning biomarkers of chronic contaminant exposure rather than direct evidence of adverse effects at the population level. Although genotoxic damage may impair cellular function, reproduction, and long-term fitness, the present study did not evaluate population abundance or demographic trends; therefore, no conclusions can be drawn regarding the status of the local mullet population. Furthermore, because *C. auratus* is a benthic-feeding species, exposure to sediment-associated contaminants may represent an important pathway in addition to dissolved metals in the water column. Consequently, future studies integrating sediment contamination, tissue bioaccumulation, biomarker responses, and population-level assessments are needed to better understand the ecological significance of the observed genotoxic effects.

A limitation of the present study is also that it was based on a single sampling site and a single reference site. Although this design was appropriate for evaluating the local impact of the Deliçay River discharge on coastal genotoxicity, future studies incorporating multiple sampling and reference sites would improve the spatial representativeness of the findings and provide a more comprehensive assessment of environmental contamination and ecological risk across the Iskenderun Gulf.

## 5. Conclusions

This study demonstrates that integrating chemical analyses with genotoxic biomarkers provides a more comprehensive assessment of the ecological effects of heavy metal contamination in coastal ecosystems. The combined application of micronucleus and comet assays enabled the early detection of biological responses that may not be evident from chemical monitoring alone. These findings highlight the importance of incorporating biomarker-based approaches into long-term environmental monitoring programs to support ecological risk assessment and improve pollution management strategies in the Iskenderun Gulf and other river-influenced coastal ecosystems. Future monitoring programs integrating chemical, biological, and ecotoxicological indicators will provide a stronger scientific basis for protecting coastal biodiversity and promoting sustainable environmental management.

## Figures and Tables

**Table 1:** Mean length and weight of *C. auratus* from study site and reference site at the northeastern Mediterranean ($\bar{x}$ ± SD) (*n* = 10).

	Winter	Spring	Summer	Autumn
Study Site				
Length (cm)	13.5 ± 1.3	14.3 ± 2.4	14.0 ± 2.5	9.0 ± 1.4
Weight (g)	19.3 ± 1.6	30.1 ± 2.2	27.5 ± 1.2	30.7 ± 2.6
Reference Site				
Length (cm)	10.8 ± 1.8	9.6 ± 1.1	16.0 ± 2.5	17.8 ± 1.5
Weight (g)	19.1 ± 1.9	17.5 ± 2.1	24.8 ± 2.5	28.8 ± 1.9

Values are presented as mean ± SD for ten *C. auratus* individuals collected from each sampling site during each sampling season (*n* = 10). A total of 80 fish specimens (40 from the reference site and 40 from the sampling site) were included in the study.

**Table 2:** The seasonally physicochemical parameters in the sampling and reference sites.

Sampling Site				
	Winter	Spring	Summer	Autumn
Temperature (°C)	16.0 ± 1.0	22.1 ± 4.1	25.0 ± 2.0	18.0 ± 1.5
D.O (mg L^−1^)	9.0 ± 1.2	5.1 ± 1.2	7.0 ± 2.5	6.3 ± 1.0
pH	6.0 ± 1.0	8.1 ± 0.1	8.0 ± 1.0	6.4 ± 1.1
Salinity (%_0_)	37.0 ± 1.2	36.0 ± 1.2	38.0 ± 1.0	35.0 ± 1.1
Electrical Con. (µS cm^−1^)	220.0 ± 0.2	310.0 ± 2.5	418.0 ± 2.1	390.1 ± 2.4
Total Dissolved solid (g L^−1^)	45.4 ± 0.1	46.6 ± 0.1	49.3 ± 0.2	47.6 ± 0.1
Reference Site				
	Winter	Spring	Summer	Autumn
Temperature (°C)	14.0 ± 1.1	23.0 ± 1.1	26.0 ± 2.1	19.0 ± 1.1
D.O (mg L^−1^)	8.3 ± 1.1	7.7 ± 1.2	7.3 ± 1.2	7.5 ± 0.3
pH	8.1 ± 1.1	8.20 ± 1.1	8.9 ± 1.1	7.9 ± 1.1
Salinity (%_0_)	36.0 ± 1.1	36.1 ± 1.1	38.1 ± 1.1	37.16 ± 1.0
Electrical Con.(µS cm^−1^)	260.0 ± 1.1	310.0 ± 1.5	415.0 ± 2.2	250.0 ± 1.5
Total Dissolved solid (g L^−1^)	43.5 ± 0.1	45.5 ± 0.1	48.8 ± 0.1	46.3 ± 0.1

Values are presented as mean ± SD (*n* = 3).

**Table 3:** Seasonal trace metal concentrations in coastal seawater samples from sampling and reference sites (µg L^−1^).

Season/Metal	Regions		TEG [20]		US EPA [21]		US EPA [22]	
Winter	Sampling Site	Reference Site	CMC	CCC	CMC	CCC	CMC	CCC
Cd **	0.117 ± 0.005	0.205 ± 0.007	<0.45 (Class 1)	0.2	40	8.8	33	7.9
Cr ^ND^	0.242 ± 0.060	0.268 ± 0.061	88	4.2	-	-	1.1	50.0
Fe ^ND^	25.286 ± 4.488	30.200 ± 5.776	101	36	-	-	-	-
Pb **	0.126 ± 0.101	0.246 ± 0.051	14	1.3	210	8.1	-	5.6
Zn ***	7.243 ± 0.693	4.685 ± 0.993	76	5.33	90	81	90	81
Spring								
Cd ^ND^	0.075 ± 0.054	0.035 ± 0.006	<0.45 (Class 1)	0.2	40	8.8	33	7.9
Cr ^ND^	0.226 ± 0.122	0.131 ± 0.067	88	4.2	-	-	1.1	50.0
Fe ^ND^	35.040 ± 3.258	23.741 ± 9.12	101	36	-	-	-	-
Pb ***	0.923 ± 0.031	0.350 ± 0.086	14	1.3	210	8.1	-	5.6
Zn **	6.901 ± 1.082	5.536 ± 1.040	76	5.33	90	81	90	81
Summer								
Cd **	0.235 ± 0.049	0.148 ± 0.012	<0.45 (Class 1)	0.2	40	8.8	33	7.9
Cr **	1.017 ± 0.177	2.088 ± 0.178	88	4.2	-	-	1.1	50.0
Fe *	50.741 ± 2.034	45.047 ± 2.01	101	36	-	-	-	-
Pb **	1.711 ± 0.506	0.548 ± 0.060	14	1.3	210	8.1	-	5.6
Zn **	10.281 ± 0.761	8.536 ± 0.247	76	5.33	90	81	90	81
Autumn								
Cd ^ND^	0.155 ± 0.058	0.097 ± 0.009	<0.45 (Class 1)	0.2	40	8.8	33	7.9
Cr ^ND^	1.503 ± 0.753	1.951 ± 0.357	88	4.2	-	-	1.1	50.0
Fe **	60.661 ± 2.411	43.279 ± 4.87	101	36	-	-	-	-
Pb **	1.738 ± 0.348	0.541 ± 0.044	14	1.3	210	8.1	-	5.6
Zn **	11.055 ± 0.431	7.113 ± 1.521	76	5.33	90	81	90	81
Annual								
Cd ^ND^	0.146 ± 0.073	0.121 ± 0.066	<0.45 (Class 1)	0.2	40	8.8	33	7.9
Cr ^ND^	0.747 ± 0.625	1.109 ± 0.969	88	4.2	-	-	1.1	50.0
Fe ^ND^	42.932 ± 14.55	35.567 ± 10.63	101	36	-	-	-	-
Pb **	1.125 ± 0.742	0.421 ± 0.144	14	1.3	210	8.1	-	5.6
Zn **	8.870 ± 2.105	6.468 ± 1.786	76	5.33	90	81	90	81

The data are shown as arithmetic mean ± standard deviation. Asterisks indicate significance levels between water samples taken from the sampling and the reference sites (ND *p* > 0.05; * *p* < 0.05; ** *p* < 0.01; *** *p* < 0.001). Turkey Environmental Guide (TEG) [20]: Specific Pollutants for Surface Water Resources and Environmental Quality Standards. CMC: Criterion maximum concentration; acceptable value in the aquatic environment. CCC: Criterion continuous concentration; the highest concentration unit to which one may be exposed. TEG: Turkey Environmental Guide; CMC: criterion maximum concentration; CCC: criterion continuous concentration. US EPA: United States Environmental Protection Agency. Water quality criteria were obtained from the National Recommended Water Quality Criteria—Aquatic Life Criteria Table (latest available edition) [21,22].

**Table 4:** Frequency (%) of micronucleus and nuclear abnormalities in peripheral blood cells of *C. auratus* from the sampling site and reference site assessed by the micronucleus test.

	Sampling Site	Reference Site
Micronucleus		
Winter ***	4.60 ± 0.08	2.53 ± 0.20
Spring ***	8.20 ± 0.17	3.80 ± 0.10
Summer ***	10.16 ± 0.15	4.76 ± 0.11
Autumn ***	3.40 ± 0.26	1.20 ± 0.10
Annual **	6.59 ± 3.13	3.07 ± 1.55
Kidney		
Winter ***	6.13 ± 0.18	2.20 ± 0.17
Spring **	8.50 ± 0.60	3.23 ± 0.05
Summer **	10.36 ± 0.32	4.46 ± 0.20
Autumn ^ND^	3.20 ± 0.17	1.40 ± 0.10
Annual ***	7.05 ± 3.09	2.82 ± 1.32
Binucleus		
Winter ***	7.76 ± 0.12	2.23 ± 0.20
Spring ***	12.20 ± 0.10	3.26 ± 0.23
Summer ***	14.20 ± 0.10	4.33 ± 0.05
Autumn **	5.33 ± 0.28	1.53 ± 0.11
Annual ***	6.62 ± 4.60	2.84 ± 1.22
Notched		
Winter ***	9.33 ± 0.12	6.16 ± 0.15
Spring ***	12.33 ± 0.15	7.03 ± 0.05
Summer ***	14.63 ± 0.20	8.76 ± 0.15
Autumn ***	8.13 ± 0.15	3.16 ± 0.15
Annual ***	11.11 ± 2.94	6.28 ± 2.34
Lobbed		
Winter **	7.60 ± 0.08	5.16 ± 0.15
Spring ***	13.16 ± 0.15	6.00 ± 0.20
Summer ***	15.43 ± 0.11	7.06 ± 0.11
Autumn ***	6.16 ± 0.28	2.16 ± 0.15
Annual ***	10.59 ± 4.42	5.10 ± 2.11
Budded		
Winter **	8.06 ± 0.09	3.067 ± 0.05
Spring ***	12.96 ± 0.25	4.40 ± 0.36
Summer **	15.33 ± 0.15	5.16 ± 0.15
Autumn ***	4.40 ± 0.36	1.90 ± 0.10
Annual ***	10.19 ± 4.90	3.63 ± 1.44

Values are presented as mean ± standard deviation (SD). Frequencies are expressed as the percentage (%) of abnormal erythrocytes relative to the total number of erythrocytes examined. Asterisks indicate significance levels between samples taken from the sampling and the reference sites (ND *p* > 0.05; ** *p* < 0.01; *** *p* < 0.001). Values represent mean ± SD (*n* = 10 fish per site per season). One thousand erythrocytes were scored for each fish.

**Table 5:** DNA damage in the gill and liver cells of *C. auratus* from sampling and reference sites analyzed by comet assay.

	Sampling Site	Reference Site
Gill		
Damage Frequency (%)		
Winter ***	32.00 ± 2.65	24.00 ± 6.08
Spring ***	40.00 ± 2.01	20.67 ± 6.35
Summer ***	46.00 ± 1.73	23.67 ± 5.77
Autumn ***	50.00 ± 3.61	20.67 ± 6.35
Annual ***	42.00 ± 7.42	22.25 ± 5.50
Arbitrary Unit (AU)		
Winter ***	64.33 ± 8.74	48.67 ± 14.57
Spring **	90.67 ± 5.51	41.00 ± 15.59
Summer ***	115.67 ± 7.02	47.33 ± 13.28
Autumn ***	121.00 ± 8.89	41.00 ± 15.59
Annual ***	97.92 ± 24.41	44.50 ± 13.14
Damage Index (DI) (%)		
Winter ***	0.64 ± 0.09	0.49 ± 0.15
Spring **	0.91 ± 0.06	0.41 ± 0.16
Summer ***	1.16 ± 0.07	0.47 ± 0.13
Autumn ***	1.21 ± 0.09	0.41 ± 0.16
Annual ***	0.98 ± 0.24	0.45 ± 0.13
Liver		
Damage Frequency (%)		
Winter ***	28.00 ± 1.01	22.67 ± 3.06
Spring ***	35.33 ± 1.15	20.67 ± 1.15
Summer ***	40.33 ± 1.59	24.00 ± 3.46
Autumn ***	45.67 ± 7.23	22.67 ± 3.06
Annual ***	37.33 ± 7.52	22.50 ± 2.71
Arbitrary Unit (AU)		
Winter ***	59.67 ± 2.08	46.00 ± 3.01
Spring **	67.33 ± 1.53	45.00 ± 3.46
Summer ***	90.00 ± 2.01	45.00 ± 1.73
Autumn ***	82.01 ± 14.01	46.00 ± 3.01
Annual ***	74.75 ± 13.87	45.50 ± 2.51
Damage Index (DI) (%)		
Winter ***	0.60 ± 0.02	0.46 ± 0.03
Spring **	0.67 ± 0.02	0.45 ± 0.03
Summer ***	0.90 ± 0.03	0.45 ± 0.02
Autumn ***	0.82 ± 0.14	0.46 ± 0.03
Annual ***	0.75 ± 0.14	0.45 ± 0.02

Values are presented as mean ± standard deviation (SD). Significance level between samples taken from the sampling and the reference sites (ND *p* > 0.05; ** *p* < 0.01; *** *p* < 0.001).

## Data Availability

The data that support the findings of this study are available on request from the corresponding author.
